# Tissue‐Resident Regulatory T Cells Expressing CD83 Maintain Local Homeostasis and Restrict Th2 Responses in Asthma

**DOI:** 10.1002/eji.202451525

**Published:** 2025-02-16

**Authors:** Anita Heiß, Susanne Krammer, Christine Kuhnt, Christina Draßner, Philipp Beck, Adriana Geiger, Stefan Schliep, Carol‐Immanuel Geppert, Alexander Steinkasserer, Andreas B. Wild

**Affiliations:** ^1^ Department of Immune Modulation Universitätsklinikum Erlangen Friedrich‐Alexander‐Universität Erlangen‐Nürnberg (FAU) Erlangen Germany; ^2^ Department of Molecular Pneumology Universitätsklinikum Erlangen Friedrich‐Alexander‐Universität Erlangen‐Nürnberg (FAU) Erlangen Germany; ^3^ Department of Dermatology Universitätsklinikum Erlangen Friedrich‐Alexander‐Universität Erlangen‐Nürnberg (FAU) Erlangen Germany; ^4^ Institute of Pathology Universitätsklinikum Erlangen Friedrich‐Alexander‐Universität Erlangen‐Nürnberg (FAU) Erlangen Germany

**Keywords:** asthma, CD83, regulatory T cells, Th2 response, tissue homeostasis

## Abstract

Non‐lymphoid tissue Tregs (NLT‐Tregs) are critical for tissue homeostasis, inflammation control, and induction of tissue repair. Recent single‐cell RNA sequencing data identified the expression of CD83 as part of an NLT‐Treg signature, which is an essential molecule for the stability and differentiation of lymphoid Tregs. However, the biological significance of CD83 expression for NLT Tregs has not yet been elucidated. The present study explores for the first time the role of CD83 expression by lung‐resident Tregs in the steady state and during asthma to understand its importance in barrier tissues. We evaluated the effect of Treg‐specific CD83 deletion (CD83cKO) on the lung‐resident T‐cell compartment and cytokine profile. CD83‐deficient lung Tregs are less differentiated but more activated, resulting in unrestrained T‐cell activation. Further, CD83cKO mice were challenged in an asthma model and showed an accelerated disease progression, driven by Th2‐biased T‐cell responses. CD83cKO Tregs exhibited enhanced responsiveness to IL‐4, leading to insufficient control of Th2‐differentiation from naïve T cells. These findings underscore the pivotal role of CD83 in the NLT‐Treg‐mediated modulation of Th2 responses. Overall, our results highlight CD83 as a key player in tissue homeostasis and inflammatory responses, suggesting potential therapeutic implications for inflammatory disorders such as asthma.

## Graphical abstract

Under steady‐state conditions, lung‐resident T cells from Treg‐specific mice (CD83cKO) are highly activated and secrete higher amounts of cytokines. During asthma, Th2‐responses derail in CD83cKO, leading to eosinophilia and lung damage. This effect relies on a destabilizing effect of IL‐4 on Tregs from CD83cKO mice.

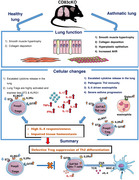

**Abbreviations**
AHR: airway hyperresponsiveness
BALF: bronchoalveolar lavage fluids
LT: lymphoid tissues
Mch: methacholine
NLT: non‐lymphoid tissues
OVA: ovalbumin
Penh: enhanced pause
Treg: regulatory T cells

## 1 Introduction

Regulatory T cells (Tregs) are crucial for suppressing inflammation, preventing autoimmunity, and promoting tissue homeostasis and repair. Tregs are categorized into thymic/natural Tregs (nTregs) and inducible/peripheral Tregs (pTregs), with nTregs developing in the thymus and pTregs arising from CD4^+^ naïve T cells in peripheral tissues under specific cytokine signals (IL‐2, TGFβ) [1]. During maturation, nTregs migrate to secondary lymphoid tissues (LTs) and later to non‐lymphoid tissues (NLTs) like skin, lungs, and colon, where they express tissue‐specific markers such as ST2 and KLRG1, and play a significant role in maintaining tissue homeostasis, limiting inflammation and allergic immune responses [2]. KLRG1 supports late Treg differentiation and suppressive functions at inflammation sites, while ST2^+^ Tregs are activated by IL‐33 to regulate inflammation via Th2‐skewed pathways [2, 3, 4, 5, 6, 7, 8]. Furthermore, ST2^+^ Tregs are highly activated and strongly suppressive and show a Th2‐biased phenotype by expressing GATA3 and producing Th2 cytokines [3, 7, 9].

Identifying tissue‐specific Treg markers is essential for understanding their specialized roles. Recent scRNA‐Seq studies showed elevated CD83 expression in NLT‐Tregs from the skin and colon compared with lymphoid Tregs [10]. Based on our earlier study demonstrating that deleting CD83 in Tregs (CD83cKO) disrupts their stability and differentiation, we were particularly interested in the role of CD83 especially in NLT Tregs. In this study, CD83cKO Tregs had reduced expression of ST2 and KLRG1 and showed impairing gut homing, which exacerbates inflammation in colitis models [11].

Given the altered phenotype of CD83cKO cells with regard to NLT Treg markers, we propose a crucial role of CD83 expression in Tregs involved in the homeostasis of tissues, especially of the ones with barrier functions. Thus, we investigated the phenotype of CD83cKO mice with a special focus on the lungs. Our analyses revealed that lung‐resident Tregs displayed impaired late differentiation in CD83cKO mice, were highly activated, and were less able to prevent overshooting cytokine responses. In a lung inflammation model for asthma, CD83cKO mice exhibited increased Th2 inflammation, eosinophilia, and airway hyperresponsiveness (AHR). Mechanistically, IL‐4 stimulation causes a dedifferentiation of CD83cKO Tregs, enhancing Th2 differentiation and proliferation. These findings highlight the critical role of CD83 in maintaining tissue homeostasis and preventing pathogenic Th2 responses.

## 2 Results

### 2.1 CD83 Mitigates Activation Status of Lung Tissue Resident Tregs and Restrains Effector T Cell Activation

**FIGURE 1:**
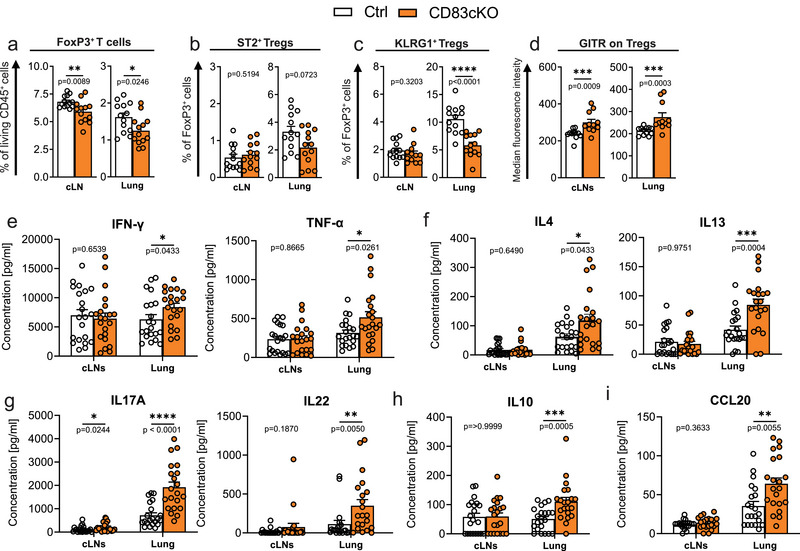
CD83 mitigates activation status of lung tissue‐resident Tregs and restrains effector T cell activation. FACS analysis of Tregs from cervical lymph node (cLN) and lungs of 8–12‐week‐old CD83cKO and control mice. (A) Frequencies of FoxP3^+^ Tregs among all living CD45^+^ in the respective organs. (B, C) Frequency of ST2^+^ and KLGR1^+^ cells among Tregs. (D) Surface receptor expression of ST2, KLRG1, and GITR on Tregs. (Ctrl *n* = 8–13, CD83cKO *n* = 8–13, pool of three independent experiments). (E–I) Lung and cervical lymph node (cLNs) derived lymphocytes from 8 to 12‐week‐old mice were stimulated for 48 h with anti‐CD3/anti‐CD28 and cytokine and chemokine secretion was measured (Ctrl *n* = 20–21, CD83cKO *n* = 20–21, pool of six independent experiments). In all graphs, data are represented as mean ± SEM and a two‐tailed Mann–Whitney *U*‐test was used to analyze the data.

In our previous study, CD83cKO mice showed a reduced presence of FoxP3^+^ Tregs in both lymphoid tissues (LTs) and the intestinal lamina propria, impairing the resolution of intestinal inflammation [11]. Since both the gut and lungs are critical sites of immune‐environment interactions, tissue‐resident Tregs of both organs are essential for maintaining tolerance. To further explore the role of CD83 in barrier tissues, we analyzed the frequency and phenotype of lung Tregs in CD83cKO mice. In concordance with our older data, Tregs were significantly reduced in the cervical lymph nodes (cLNs) and lungs of CD83cKO mice (Figure 1A). Lung Tregs showed a trend toward decreased ST2 expression and we observed a marked reduction in KLRG1^+^ Tregs (Figure 1B,C; Figure S1), a change which was specific to nonlymphoid tissue (NLT) Tregs, as no genotype differences were present in cLN‐derived cells (Figure S2A). Expression of ICOS and CD25 was unaffected, but GITR, a marker of heightened T cell activation, was significantly upregulated in all tissues (Figure 1D; Figure S2B).

The reduction in KLRG1^+^ Tregs, coupled with elevated GITR levels, suggested impaired suppression of effector T‐cell activation. Upon stimulation of lung or cLN cells with anti‐CD3/anti‐CD28, lung‐derived lymphocytes from CD83cKO mice exhibited elevated cytokine release, including cytokines associated with Th1, Th2, and Th17 responses (Figure 1E–G). Furthermore, IL‐10 was significantly increased (Figure 1H) and the chemokine CCL20 (Figure 1I). This heightened response was more pronounced in lung cells than in cLNs, except for IL‐17A (Figure 1G). These findings indicate that CD83 deletion in Tregs compromises their ability to regulate tissue‐resident T‐cell activation, leading to excessive cytokine and chemokine production and promoting lung inflammation. This hypothesis was further examined using an allergic asthma model to assess its in vivo relevance.

### 2.2 CD83cKO Mice Show Exacerbated Airway Hyperresponsiveness and Enhanced Eosinophilic Inflammation

**FIGURE 2:**
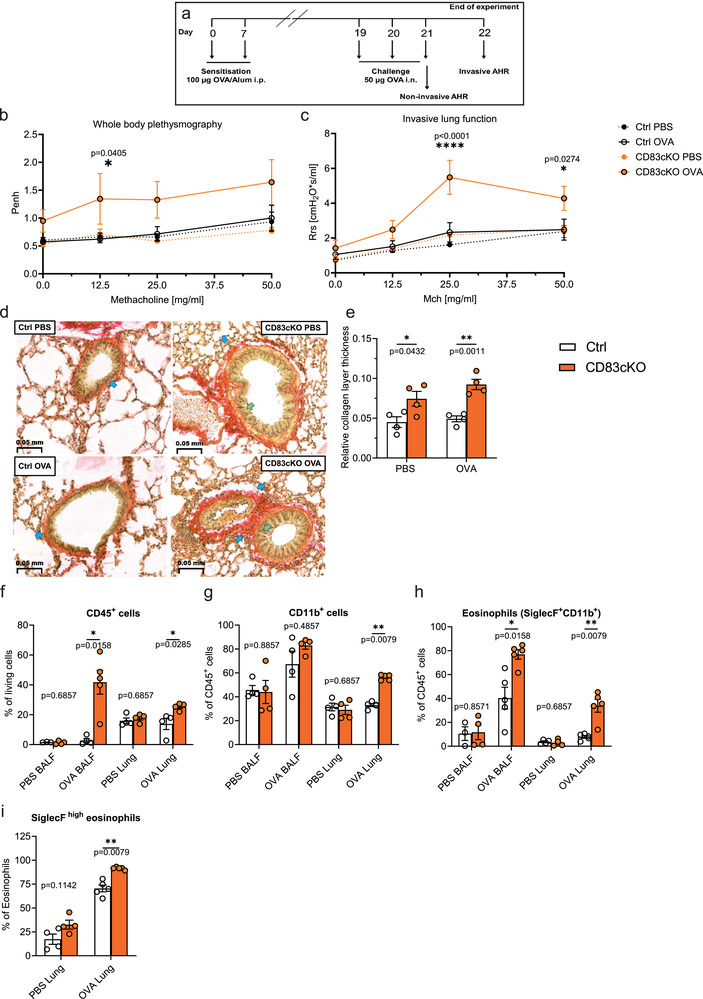
CD83cKO mice showed increased AHR and pathological changes in lung morphology. (A) Graphical overview experimental procedure for the in vivo allergic asthma mouse model. (B) Noninvasive AHR measurement by whole‐body plethysmography (Ctrl *n* = 4–5, CD83cKO *n* = 4–5, one representative experiment out of two). Enhanced pause (Penh) has been calculated at baseline and after challenge with methacholine (Mch): 0 mg/mL: *p* = 0.4926; 25 mg/mL: *p* = 0.1067; 50 mg/mL: *p* = 0.0826. (C) Invasive AHR analysis was performed and the resistance (Rrs) of the lung was calculated upon methacholine challenge: 0 mg/mL: *p* = 0.938; 12.5 mg/mL: *p* = 0.4068. (D) Representative Sirius Red stainings of lung tissue from PBS or OVA‐treated Ctrl and CD83cKO mice. Green arrows demonstrate enlarged bronchial epithelium and blue arrows indicate collagen deposition. (E) The thickness of the peribronchial collagen layer was quantified by dividing the collagen thickness beneath the bronchial epithelium by the bronchial diameter. (F–I) Flow cytometric analysis of immune cells in the bronchoalveolar lavage fluid (BALF) and lung tissue from asthmatic and control mice. (F) Frequencies of CD45^+^ immune cells, (G) total CD11b^+^ cells, as well as (H) eosinophils in the BALFs and lungs. (I) Frequencies of SiglecF^high^‐inflammatory eosinophils in lung tissue. Statistical analysis of AHR data was performed using a two‐way ANOVA test with Tukey´s correction test. Relative collagen thickness and flow data are represented as mean ± SEM and a two‐tailed Mann–Whitney *U*‐test was used for statistical analysis.

To induce asthma, mice were immunized twice with ovalbumin (OVA) in alum and challenged intranasally with OVA alone two weeks after the final immunization, while PBS‐treated mice served as controls (Figure 2A). Airway hyperresponsiveness to methacholine (Mch) was measured using both noninvasive and invasive methods. OVA‐treated CD83cKO mice showed increased enhanced pause (Penh) at 12.5 mg/mL Mch, indicating airway narrowing (Figure 2B). They also displayed significantly impaired lung function, with elevated resistance and elastance and reduced compliance (Figure 2C; Figure S3A,B).

Histological analysis of lung tissues from asthma‐induced mice revealed increased collagen deposition around small bronchioles in CD83cKO mice, even in PBS‐treated controls, which became more pronounced after OVA treatment (Figure 2D; Figure S4). The semiquantitative evaluation confirmed significantly thicker collagen in CD83cKO lungs, indicative of pathological changes (Figure 2E). Additionally, OVA‐treated CD83cKO mice exhibited notable peribronchial and perivascular cellular infiltrates (Figure S5).

Inflammatory eosinophil infiltration, a hallmark of OVA‐induced asthma [12], was assessed in bronchioalveolar lavage fluids (BALFs) and lung tissues. OVA‐treated CD83cKO mice showed a dramatic increase in CD45^+^ immune cells in BALFs (Figure 2F), which consisted primarily of CD11b^+^/SiglecF^+^ eosinophils (Figure 2G,H). Similarly, the lungs of these mice contained significantly more eosinophils, particularly the inflammatory SiglecF^high^ subset (Figure 2I; Figure S6). We did not observe changes in immune cell composition in spleens or cLNs, confirming the lung‐specific effects of CD83 deletion (Figure S7A,B).

### 2.3 Exacerbated Asthmatic Disease in CD83cKO Mice Is Promoted by Insufficient Control of Th2 Immunity

**FIGURE 3:**
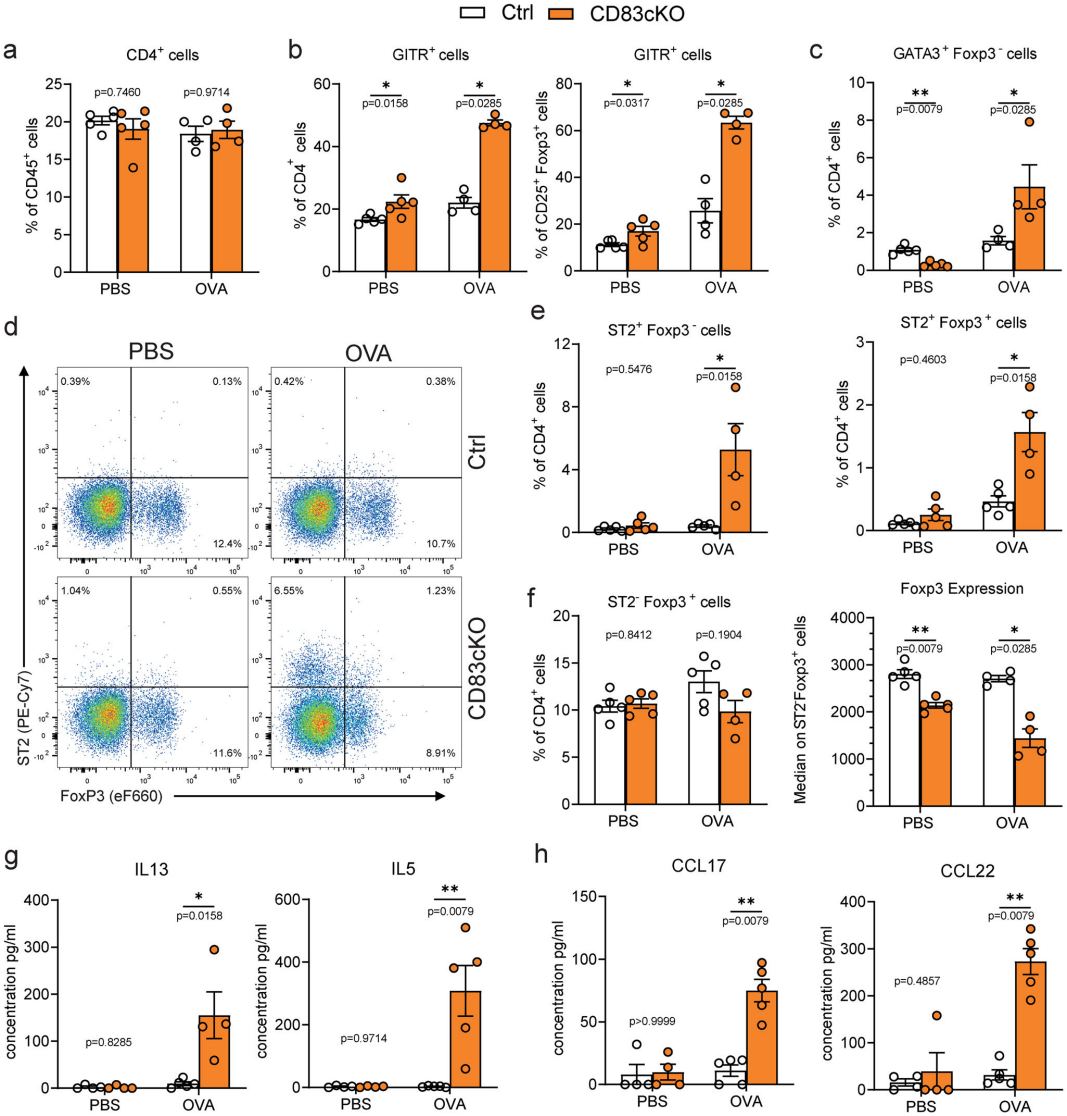
Asthmatic lungs derived from CD83cKO mice show enhanced Th2 effector T‐cell responses and a highly activated phenotype. (A–F) Flow cytometric analysis of lung‐resident CD4^+^ T cells and Tregs from asthmatic and control mice: (A) CD4^+^ T‐cell frequencies among all CD45^+^ cells. (B) Percentage of GITR^+^ cells among CD4^+^ T cells and Tregs. (C) Th2 effector cells (Foxp3^−^GATA3^+^) frequencies in the CD4^+^ T cell population. (D) Representative graphs of ST2 and FoxP3 populations among CD4^+^ cells. (E) Analysis of ST2^+^ Foxp3^−^ effector cells and ST2^+^Foxp3^+^ Tregs among CD4^+^ T cells. (F) Percentages of St2^−^Foxp3^+^ Tregs among lung‐resident T cells, and median expression of FoxP3 on tissue‐resident Tregs. (G) Cytokine and (H) chemokine analysis of lung lymphocytes re‐stimulated with OVA for 24 h. In all graphs, data are represented as mean ± SEM and a two‐tailed Mann–Whitney *U*‐test was used to analyze the data (Ctrl *n* = 4–5, CD83cKO *n* = 4–5, one representative experiment out of two).

In addition to eosinophils, we analyzed CD4^+^ T‐cell populations in various tissues. CD83cKO mice showed no differences in total CD4^+^ T cell frequencies compared with controls (Figure 3A; Figure S8A). However, lung CD4^+^ and CD25^+^FoxP3^+^ Tregs from CD83cKO mice exhibited significantly elevated GITR expression, even before OVA treatment, with further increases following treatment (Figure 3B; Figure S8B–D). Elevated GITR levels can enhance Th2 cell activity, exacerbating airway inflammation [13]^,^ [14]. Consistent with this, OVA‐treated CD83cKO mice showed a significant increase in GATA3^+^FoxP3^−^ Th2 cells (Figure 3C; Figure S3E). Analysis of additional Th2 markers revealed increased frequencies of ST2^+^ subsets in both FoxP3^+^ Tregs and FoxP3^−^ effector cells (Figure 3D,E). Remarkably, lung Tregs from CD83cKO mice had reduced FoxP3 expression even without OVA challenge (Figure 3F). Since ST2 is the receptor for IL‐33, an alarmin that supports tissue homeostasis and expands NLT‐Tregs during inflammation, serum IL‐33 levels were measured. OVA‐treated CD83cKO mice displayed a trend toward elevated IL‐33 levels, indicating heightened inflammation (Figure S9).

Ex vivo restimulation of lung immune cells with OVA further highlighted the inability of CD83‐deficient Tregs to regulate Th2 responses. While PBS‐treated cells showed no reaction, OVA‐challenged CD83cKO cells released significantly more Th2‐related cytokines like IL‐5 and IL‐13 and the chemokines CCL17 and CCL22 (Figure 3G,H). These findings indicate that CD83‐deficient Tregs, characterized by reduced terminal differentiation and elevated GITR expression, fail to control Th2 activity, leading to severe eosinophilia and an exacerbated disease course in allergic asthma.

### 2.4 CD83‐Deficient Tregs Fail to Confine Th2 Differentiation and Proliferation

**FIGURE 4:**
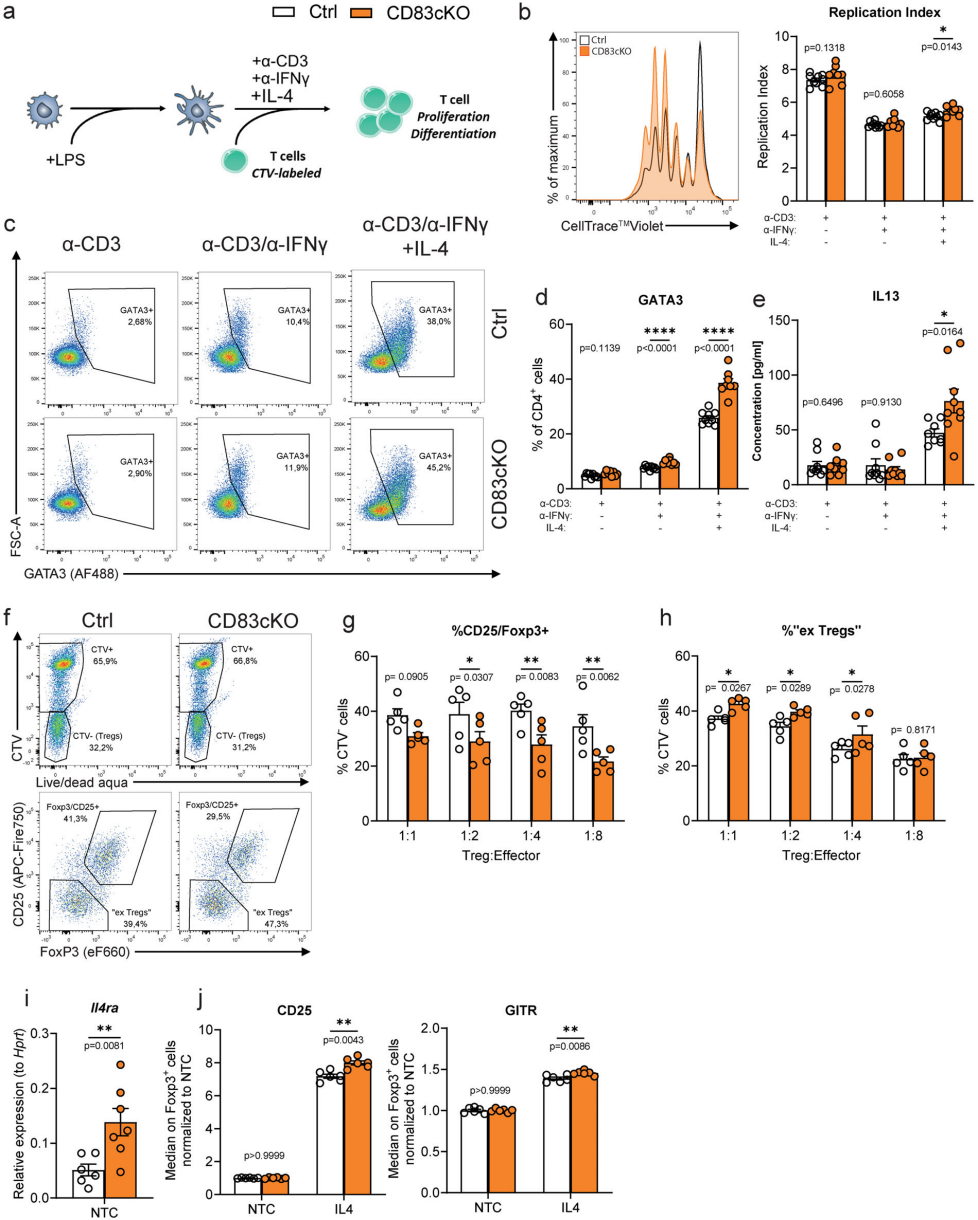
IL‐4 dependent destabilization of CD83cKO Tregs leads to insufficient control of Th2 cell differentiation. (A) Schematic overview of DC/T cell coculture: LPS‐stimulated DCs are co‐cultured with CellTrace Violet (CTV)‐labeled CD4^+^ T cells from wild‐type or CD83cKO mice in the presence of stimuli (anti‐CD3/anti‐IFNγ/IL‐4) for 4 days. (B) Representative histogram showing the proliferation of CD83CKO and control cells and the evaluation by the replication index. (C) Representative plots of GATA3 expression in CD4^+^ T cells under different cultivation conditions. (D) Frequency of GATA3^+^ T cells amongst CD4^+^ T cells. (E) Assessment of IL‐13 production in supernatants of co‐cultures (*n* = 9, pool of three independent experiments). (F) Representative dot plots of CD4^+^ T cells (upper panel) and (lower panel) from the suppression assay. Percentages of (G) CD25^+^/FoxP3^+^ cells and (H) ex‐Tregs among CTV^−^ cells (*n* = 5, pooled from two independent experiments). (I) Gene expression analysis of *Il4ra* in sorted Tregs (CD3^+^CD4^+^YFP^+^) from spleens of Ctrl and CD83cKO mice. (J) Median fluorescence of CD25 and GITR on Foxp3^+^ Tregs after stimulation with IL‐4 in relation to nontreated control (NTC) (*n* = 6, pool of three independent experiments). Data are represented as mean ± SEM and a two‐tailed Mann–Whitney *U*‐test was used for analysis.

To scrutinize the impaired ability of CD83cKO Tregs to suppress Th2 responses, we conducted Th skewing experiments to examine CD4^+^ T cell proliferation and differentiation under Th2 conditions. CD4^+^ T cells from CD83cKO or control mice were labeled with CellTrace Violet and co‐cultured with mature dendritic cells (DCs; Figure 4A). The cultures were set up as follows: (1) anti‐CD3 antibodies to stimulate T‐cell proliferation, (2) anti‐IFNγ antibodies to inhibit Th1 differentiation, and (3) IL‐4 to promote full Th2 differentiation. In these co‐culture experiments, we observed that Treg frequencies remained unchanged between both groups, except for an increase in the anti‐CD3/anti‐IFNγ condition (Figure S11A). While T‐cell proliferation was unaffected in anti‐CD3 or anti‐CD3/anti‐IFNγ conditions, proliferation significantly increased under full Th2 conditions (Figure 4B). GATA3 expression, the master transcription factor of Th2 differentiation, was unaltered in anti‐CD3‐only co‐cultures but increased when IFN‐γ signaling was blocked (Figure 4C,D). This effect was further amplified upon the addition of IL‐4, leading to a robust induction of Th2 differentiation (Figure 4D). Consistently, IL‐13 levels were markedly elevated in the supernatants of CD83cKO‐derived T cells cultured under full Th2 conditions (Figure 4E). Moreover, IL‐4 also enhanced the surface expression of GITR and CD25 on these cells (Figure S11B). These findings demonstrate that CD4^+^ T cells from CD83cKO mice have an enhanced propensity for Th2 differentiation, which is fully unleashed upon IL‐4 stimulation.

### 2.5 Increased Susceptibility of CD83cKO Tregs IL‐4 Stimulation Deteriorates Their Stability

We know from our previous study, that CD83cKO Tregs, while less functional in vivo, exhibited comparable suppressive capacity to wildtype Tregs in vitro [11]. As we have unraveled a profound differential effect of IL‐4 on CD4^+^ T cells from CD83cKO mice, we next assessed CD83‐deficient Tregs in a suppression assay under Th2 conditions. We co‐cultured YFP^+^ Tregs with CTV‐labeled YFP^−^ effector T cells at decreasing ratios, using mature DCs as stimulators, together with either anti‐CD3 alone or a full Th2‐Mix. CD83cKO Tregs suppressed effector proliferation similarly to controls as well as cellular activation, as indicated by reduced CD25 expression on effector T cells (Figure S12A,B). However, analysis of CTV^−^ cells (which represent unlabeled YFP^+^ T cells, i.e., Tregs) revealed a significant reduction in CD25^+^/FoxP3^+^ cells among CD83cKO Tregs and enrichment of ex‐Tregs (former Tregs lacking CD25 and FoxP3) in Th2‐Mix conditions, but not with anti‐CD3 alone (Figure 4F–H; Figure S12C,D). These findings align with reports that IL‐4 can inhibit iTreg differentiation and promote the conversion of Tregs to exFoxp3‐Th2 cells [15, 16]. Indeed, sorted CD83cKO Tregs exhibited elevated *Il4ra* transcripts, suggesting increased IL‐4 responsiveness (Figure 4I). As expected from literature reports [17], FoxP3 expression was stabilized and increased when Tregs were stimulated with IL‐4, regardless of the genotype (Figure S12E). Interestingly, CD83cKO Tregs showed heightened CD25 and GITR expression upon treatment with IL‐4, indicative of enhanced activation (Figure 4J). Additional activation markers, including ICOS and CD69, were not influenced by the Treg‐specific CD83 deletion (Figure S12F,G). Blocking IL‐4 signaling reversed these effects (Figure 12H), confirming the IL‐4 dependency of this highly active phenotype of CD83cKO Tregs.

Collectively, our data disclosed a novel role of CD83 in NLT‐Tregs since its deletion heightens IL‐4 responsiveness, destabilizing Tregs and undermining their ability to suppress Th2 responses. This contributes to an activated, proinflammatory Treg phenotype, disrupting tissue homeostasis and exacerbating Th2‐driven asthma symptoms. These findings highlight the pivotal regulatory role of CD83 in Treg biology and function.

## 3 Discussion

Most NLT‐Tregs, especially those at barrier sites like the skin, colon, and lungs, are primed in lymphoid tissues before migrating to the periphery. During this migration, they undergo specific transcriptomic changes and adapt to their local environment, including upregulation of markers such as KLRG1, ST2, and GATA3. These adaptations enable their differentiation into effector Tregs, which play a critical role in suppressing pathogenic responses at sites of inflammation or injury [2, 10]. Single‐cell sequencing has shown that CD83 is highly expressed in Tregs residing in barrier tissues [10], supporting our previous findings that deletion of CD83 in Tregs results in a proinflammatory phenotype, impaired differentiation, and compromised resolution of inflammatory events [11]. Additionally, reduced ST2 and KLRG1 expression in splenic CD83cKO Tregs suggests a pronounced impact of CD83 deletion on tissue‐resident Tregs.

In the present study, lung T cells from CD83cKO mice exhibited a marked increase in cytokine secretion upon stimulation with anti‐CD3/CD28, an effect specific to NLT T cells and absent in cLNs. Among the dysregulated cytokines, those associated with Th17 responses were most prominently increased. Lung lymphocytes from CD83cKO mice also secreted more CCL20, a chemokine critical for recruiting dendritic cells (DCs) and Th17 cells. IL‐17, while essential for lung tissue homeostasis and bacterial clearance, can cause fibrosis and pathological airway remodeling when excessively produced [18]. Indeed, unchallenged CD83cKO mice displayed morphological changes in their lungs, including epithelial hyperplasia, smooth muscle hypertrophy, and increased collagen deposition. This indicates that CD83‐deficient Tregs have trouble maintaining tissue integrity, even in the absence of inflammation. Additionally, CD83cKO mice secreted elevated IL‐10, an anti‐inflammatory cytokine that also can enhance Th2 responses in a house dust mite‐driven asthma model [19].

We further revealed that CD83cKO Tregs expressed significantly higher levels of GITR, a co‐stimulatory molecule associated with Treg plasticity and a shift toward Th9 and Th2 phenotypes [13, 20–22]. GITR signaling has been linked to reduced Treg stability and suppressive capacity [23], exacerbating autoimmune diseases, and fostering Th2‐driven asthma [13, 22, 24]. This perfectly aligns with our in vivo data in the OVA‐induced asthma model, where CD83cKO mice developed more severe Th2‐driven symptoms, including increased eosinophilia and airway remodeling. These mice also displayed elevated ST2 expression on lung T cells and a trend toward higher IL‐33 levels in their sera. IL‐33 stimulation has been shown to impair Treg function, promote Th2‐like Treg phenotypes, and drive IL‐5 and IL‐13 production—key mediators of eosinophil recruitment and airway inflammation [25]. Lung lymphocytes from CD83cKO mice secreted significantly more IL‐5 and IL‐13 upon OVA restimulation, further emphasizing the inability of CD83‐deficient Tregs to suppress Th2 responses effectively. While IL‐13 is known to induce tissue remodeling, airway hyperresponsiveness, and mucus secretion in the lung [26, 27], IL‐5 is a key stimulus for the recruitment and survival of eosinophils [28]. Concomitantly, CD83cKO mice had increased collagen deposition around the bronchi as well as massive eosinophilia in the lung tissue and the BALF. Additionally, CD83cKO mice exhibited higher levels of chemokines like CCL17 and CCL22, which are essential for recruiting Th2 cells to inflamed lung tissue [29, 30]. Together, these findings underscore the failure of CD83‐deficient Tregs to regulate Th2 immunity, resulting in exacerbated disease progression.

Despite retaining their suppressive capacity in vitro, we already have demonstrated that CD83cKO Tregs showed a context‐dependent functional impairment in vivo [11]. In the present study, we revealed that CD83cKO was less able to suppress Th2 differentiation, which became more evident as Th1 commitment was inhibited. CD83cKO Tregs exhibited increased expression of IL‐4Rα, rendering them more responsive to IL‐4 and promoting elevated CD25 and GITR levels. IL‐4 destabilized these Tregs, aligning with studies showing that STAT6‐mediated Foxp3 inhibition drives pathogenic Th2 responses [8, 31].

Interestingly, a soluble form of CD83 (sCD83) with well‐described immunomodulatory functions [32] has been shown to induce apoptosis in Th2 cells in models of allergic rhinitis [33]. This highlights a potential immunomodulatory role for sCD83 in controlling Th2 responses. Since activated Tregs upregulate and stably express CD83 [34], this pathway adds complexity to Treg‐mediated immune regulation.

In summary, our findings describe a previously unknown crucial role of CD83 in the suppressive function of NLT‐Tregs and the maintenance of tissue homeostasis. Its deletion in Tregs results in a hyperactive and proinflammatory phenotype, impairing their ability to suppress Th2 responses and driving IL‐4‐dependent pathogenic immunity. These findings reveal a previously unknown role for CD83 in regulating Treg function and controlling inflammation in barrier tissues.

## 4 Methods

### 4.1 Animals

Mice were maintained on the C57BL/6 strain background. Floxed CD83 (CD83^fl/fl^) animals were generated in our laboratory as described previously [35]. Animals were provided by A. Rudensky (University of Washington, Seattle, Washington, USA). For Treg‐specific depletion of the CD83 gene, CD83^fl/fl^ animals were crossed with Foxp3‐Cre mice (CD83cKO). The Foxp3‐Cre were used as controls (Ctrls) in all experiments. In all experiments, we used an equal distribution of age‐matched male and female mice. Mice were housed in a controlled environment with specific pathogen‐free conditions, including a temperature of 22°C and humidity between 40% and 50%, following a 12 h light–dark cycle with ad libitum access to food and water. All animal care and experimental procedures in this study followed the guidelines of the European Community Standards for Laboratory Animal Care and were approved by the local ethics committee (Administration of Lower Franconia; reference number 55.2‐2532‐2‐633).

### 4.2 Allergic Asthma In Vivo Mouse Model Induction

For the in vivo ovalbumin‐induced allergic asthma model mice were sensitized by intraperitoneal (i.p.) injections of 100 µg OVA (Calbiochem,) complexed with 10% aluminum potassium sulfate solution (Sigma Aldrich) on days 0 and 7. On days 19, 20, and 21, animals were challenged with intranasal application of 50 µg OVA dissolved in PBS. On day 21, whole‐body plethysmography using the Buxco system (Data Sciences International (DSI) is performed for noninvasive airway hyperresponsiveness measurement. The mice are challenged by increasing doses of methacholine in order to obtain the parameter‐enhanced pause (Penh). On day 22, an invasive AHR provocation test was carried out to analyze the lower respiratory system resistance (Rrs), elastance (Ers), and compliance (Crs) following the methacholine challenge. The experimental animals were anesthetized (Ketamine, Medetomidine) and underwent a tracheotomy to mechanically ventilate them with a blunt cannula via the flexiVent device (SCIREQ Inc.). Finally, BAL was performed and mice were sacrificed by cervical dislocation under ongoing anesthesia to isolate heart blood as well as the different tissues for the subsequent assays [36].

### 4.3 Collection of Single‐Cell Solutions

#### 4.3.1 Spleen

For isolation of splenic cells, the spleen is isolated from the mouse and ground between the frosted ends of microscope slides. The resulting cell suspension is transferred through a 70 µm cell strainer into a 50 mL tube. After lysis of erythrocytes, cells are counted and used according to subsequent procedures.

#### 4.3.2 Lymph Nodes

Lymph nodes are isolated from the mouse and ground between the frosted ends of microscope slides. The resulting cell suspension is transferred through a 70 µm cell strainer into a 50 mL tube, and the cell count is determined.

#### 4.3.3 Lung

After removal from the animal, lungs are cut into small pieces and transferred into a C‐Tube (Miltenyi Biotec) containing a 2 mL digestion solution that consists of FCS (5%, Thermo Fisher Scientific), Collagenase‐D (1 mg/mL, Roche) and DNase I (0.5 mg/ml, Applichem) in DMEM/F12 (Thermo Fisher Scientific). Dissociation is performed with the gentleMACS Octo Dissociator (Miltenyi Biotec) and the program 37C_m_LDK1. The resulting homogenate is passed through a moistened 70 µm Cellstrainer. Subsequently, erythrocyte lysis is performed with ACK‐Lysis‐Buffer and the cell suspension is subsequently counted and subjected to flow cytometry analysis (FACS) for further characterization and investigation.

### 4.4 Flow Cytometry Analysis and Cell Sorting

Single‐cell suspensions from different tissues were used for cell surface staining using flow cytometry employed standard procedures and the following conjugated antibodies and reagents: Live/Dead (ThermoFisher Scientific); CD3 (17A2; 1:200), CD4 (RM4‐5; 1:200), CD25 (PC61; 1:100), CD45 (30‐F11; 1:200), ST2 (DIH4; 1:100), KLRG1 (2F1; 1:200), GITR (DTA‐1; 1:100), CD11b (M1/70; 1:400), I‐A/I‐E (M5/114.15.2; 1:200), SiglecF (S17007L; 1:100), Ly6G (1A8; 1:200), (Biolegend). Intracellular staining for Foxp3 (FJK‐16s; 1:100) and GATA3 (TWAJ; 1:100) was analyzed using the Foxp3/transcription factor staining buffer set from eBioscience. Single‐cell suspensions were incubated with appropriate fluorochrome‐conjugated monoclonal antibodies and measured on FACSCanto II and FACSFortessa (BD Biosciences) analyzing the data with FlowJo v10 (Tree Star). Sort experiments of CD3^+^ CD4^+^ YFP^+^ Tregs were performed on FACS Aria.

### 4.5 Restimulation of cLN and Lung Derived Lymphocytes

Cells were isolated from the cLNs and the lung. Cells (1 × 10^5^) were incubated for 48 h with anti‐CD3 (1 µg/mL) and anti‐CD28 (1 µg/mL) or for 24 h with OVA (500 µg/mL) in 100 µL RPMI1640 medium (Anprotec), containing 10% fetal bovine albumin (FCS, Merck), 1% penicillin/streptomycin/l‐glutamine solution (Sigma) and 0.5 µM β‐mercaptoethanol (Roth) (R10 medium) in a 96‐well U‐bottom plate. Supernatants were collected to determine T helper cytokine and proinflammatory chemokine levels using cytometric bead arrays (BioLegend) according to the manufacturer's instructions.

### 4.6 Lung Histology

After the lung was removed, a piece was obtained from the left lobe and was fixed in 4 % paraformaldehyde, which was then embedded in paraffin. Subsequently, the tissue was sectioned using microtome into 5 µm thick serial sections for hematoxylin and eosin (H&E) and Sirius‐red staining. Peribronchial collagen was quantified by measuring the collagen thickness (µm) beneath the bronchial epithelium and normalized by dividing by the bronchial diameter (µm) [37]. Stainings were analyzed via slide viewer 2.7.0.191696 software (3DHistech), 20× magnification, scanned by slides scanner P1000 (3DHistech).

### 4.7 Serum Collection

Whole blood was collected from the heart on day 22 of the allergic asthma experiments and transferred to Microtainer Blood Collection (BD). After incubation at room temperature (RT) for 30 min, blood was centrifuged (12,500 rpm, 5 min, RT) and serum was frozen at −20°C for enzyme‐linked immunosorbent assay (ELISA) analysis.

### 4.8 Bronchoalveolar Lavage Fluid

BALF was collected on day 22 by injecting and aspirating twice 0.8 mL Sterofundin (B. Braun Melsungen) intratracheally in each mouse. Afterward, BALF was centrifuged at 1500 rpm for 5 min and the supernatants were frozen at −20°C for ELISA analysis. The remaining cell pellet was resuspended in PBS and used for flow cytometric analysis as described below.

### 4.9 Enzyme‐Linked Immunosorbent Assay

Serum levels of IL‐33 were determined by using a commercial kit for mouse‐IL‐33 according to the manufacturer's protocol (eBioscience).

### 4.10 Generation and Stimulation of Bone Marrow‐Derived Dendritic Cells

The generation of bone marrow‐derived dendritic cells followed the methodology previously described by our group [38]. Wildtype DCs were treated with LPS (10 ng/mL, Sigma Aldrich) for 3 h at 37°C, 5% CO_2_, and used for co‐culture experiments.

### 4.11 DC–T Cell Co‐Cultures

CD4^+^ T cells were isolated using the Mouse CD4^+^ T cell Isolation Kit, in accordance with the provided protocol (Miltenyi Biotec) from the spleen and cLNs followed by CTV (CellTraceViolet, Thermo Fisher Scientific) labeling according to the manufacturer´s protocol. Afterward, 1 × 10^5^ CTV‐labeled CD4^+^ T cells were co‐cultured in a 96‐well flat bottom plate at a ratio of 20:1 with LPS‐matured DCs (5 × 10^3^). Additionally, three different stimulation mixes were added for 4 days: (1) anti‐CD3 only (2.5 ng/mL, 17A2, Biolegend), (2) anti‐CD3 and anti‐IFNγ (10 µg/mL, XMG1.2, Biolegend), and (3) anti‐CD3/anti‐IFNγ and IL‐4 (10 ng/mL, Miltenyi Biotec). After co‐culture for 96 h, cells were harvested, stained with mAbs, and analyzed by flow cytometry. The replication index was calculated using FlowJo's built‐in proliferation analysis tool. Supernatants and mRNA were isolated as well and stored at −80 for further investigation.

### 4.12 Suppression Assay

For the suppression assay, the above‐mentioned protocol was modified: Single cell suspension from the spleen and cLNs were sorted for CD4^+^/CD25^+^/YFP^+^ cells (Treg) and CD4^+^/CD25^−^/YFP^−^ T effector cells on a FACS ARIA II cell sorter. Effector cells (pooled from Ctrl and CD83cKO mice) were labeled with CTV and co‐cultivated with LPS‐matured DCs. Tregs were added in decreasing amounts and cultures were stimulated with either anti‐CD3 alone or the complete Th2‐Mix. Cultures were harvested after 96 h and analyzed via flow cytometry. Suppressive capacity was calculated by dividing the replication index of Treg‐co‐cultures by the replication index of effector cells only.

### 4.13 qPCR

Sorted YFP^+^ Tregs as well as co‐cultured cells were harvested and lysed with the RLTPlus buffer (Qiagen), and total RNA was isolated by using RNeasy Plus Micro Kit (Qiagen) according to the manufacturer's protocol. RNA was transcribed into cDNA using the first strand cDNA synthesis kit (Thermo Fisher Scientific). Subsequently, gene expression was determined by using 2× qPCR S'Green BlueMix (Biozym Scientific) on CFX96 Real‐Time PCR Detection System (Bio‐Rad). All primers were tested for specificity and quality according to the MIQE guidelines [39]. Sample mRNA levels were normalized to the expression of the HPRT reference gene.

Primer sequences for *Hprt*: 5′‐GTTGGATACAGGCCAGACTTTGTTG‐3′ and 5′‐GATTCAACTTGCGCTCATCTTAGGC‐3′.

Primer sequences for Il4ra: 5′‐ACGTGGTACAACCACTTCCA‐3′ and 5′‐CTGGGGTGGGAATCTGGTCC‐3′.

### 4.14 IL‐4 Restimulation of YFP^+^ Sorted Tregs

Sorted CD3^+^ CD4^+^ YFP^+^ Tregs (1 × 10^5^) were pipetted into a 96‐well U‐bottom plate in 100 µL final volume and stimulated overnight with IL‐4 (10 ng/mL, Milteny Biotec) in R10 medium. Two control conditions are also analyzed: (1) nontreated controls (NTC) with only T cell medium and (2) pretreatment with anti‐IL‐4 (1 µg/mL, Biolegend, clone 11B11) for 30 min followed by IL‐4 stimuli as described before. The next day, cells were harvested and analyzed via flow cytometry.

### 4.15 Statistics

Statistics were calculated with Prism version 10.2.0 (GraphPad) using a two‐tailed Mann–Whitney *U* comparison test or a two‐way ANOVA test with Tukey's correction test, as specified in the figure legends. All data are presented as mean ± SEM. *p*‐values less than 0.05 were considered statistically significant, with *p < 0.05, **p < 0.01, ***p < 0.001, ****p < 0.0001. Graphs without stars are considered not significant.

## Author Contributions

Andreas B. Wild and Alexander Steinkasserer conceived the project and designed the experiments. Anita Heiß, Susanne Krammer, Christine Kuhnt, Christina Draßner, Philipp Beck, Adriana Geiger, and Andreas B. Wild performed experiments. Susanne Krammer and Adriana Geiger performed the induction of allergic asthma disease and the AHR measurements. Stefan Schliep contributed to the paraffin embedding lung tissue. Carol‐Immanuel Geppert performed the H&E and Sirius red staining and critically evaluated the pathological features of the histological results. Anita Heiß and Andreas B. Wild analyzed the results and wrote the manuscript. Susanne Krammer, Carol‐Immanuel Geppert, and Alexander Steinkasserer revised the manuscript; Alexander Steinkasserer and Andreas B. Wild supervised the project and acquired the funding.

## Conflicts of Interest

The authors declare no conflicts of interest.

## Ethics Statement

All animal care and experimental procedures in this study followed the guidelines of the European Community Standards for Laboratory Animal Care and were approved by the local ethics committee. (Administration of Lower Franconia; Reference number 55.2‐2532‐2‐633).

### Peer Review

The peer review history for this article is available at https://publons.com/publon/10.1002/eji.202451525

## Supporting information



Supporting Information

## Data Availability

The data that support the findings of this study are available from the corresponding author upon reasonable request.
