# Supplementary material for: Tissue‐Resident Regulatory T Cells Expressing CD83 Maintain Local Homeostasis and Restrict Th2 Responses in Asthma
Source: Eur J Immunol. 2025 Feb 16;55(2):e202451525. doi: 10.1002/eji.202451525 (PMC11830382; doi:10.1002/eji.202451525)
Supplement: Supplementary file 1 — Supporting Information [file EJI-55-e202451525-s001.pdf]

Supplementary figure 1
Gating strategy for NLT analysis

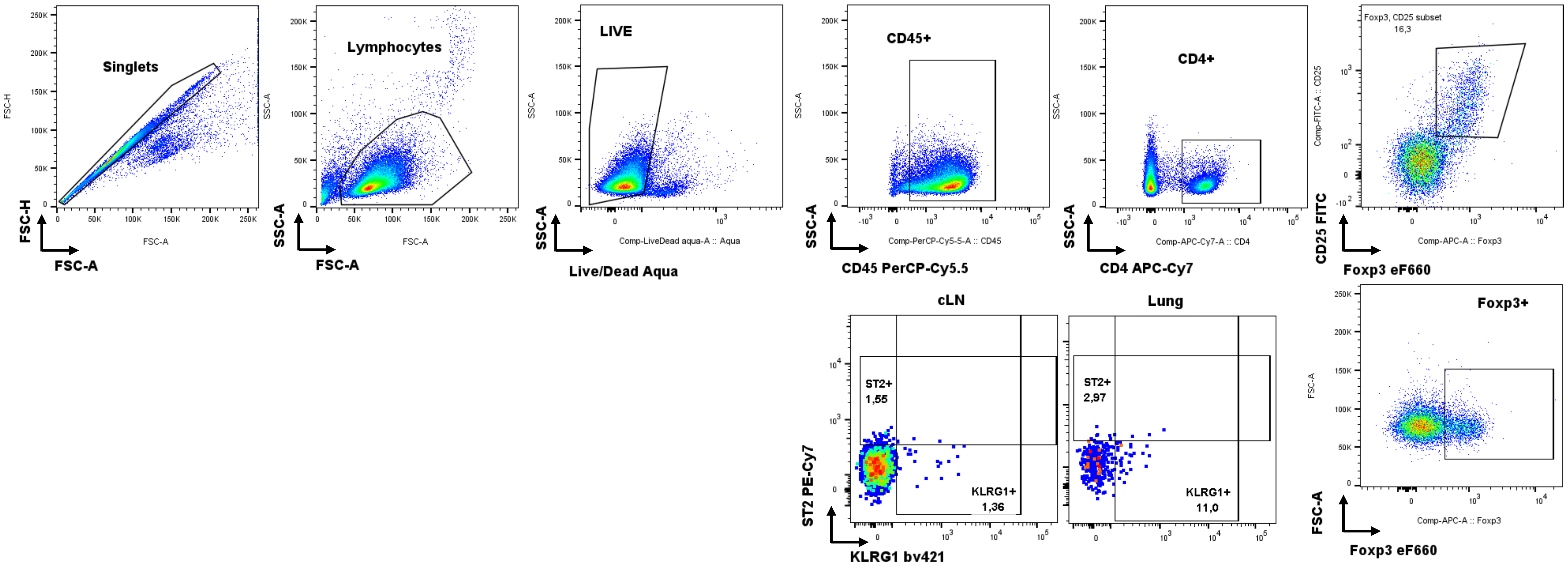

Supplementary figure 1. Flow cytometric gating strategy for the analysis of CD4+ T cells

Gating strategy to identify CD4+T-cells and regulatory T cells (CD25+ Foxp3+). Different surface receptors (CD25, ST2, GITR and KLRG1) and transcription factors (Foxp3 and GATA3) were analyzed on these subsets. Color dot plot of a representative subject. Lower panel contains staining for ST2 and KLRG1 amongst FoxP3+ cells in cLN and lungs.

Supplementary figure 2

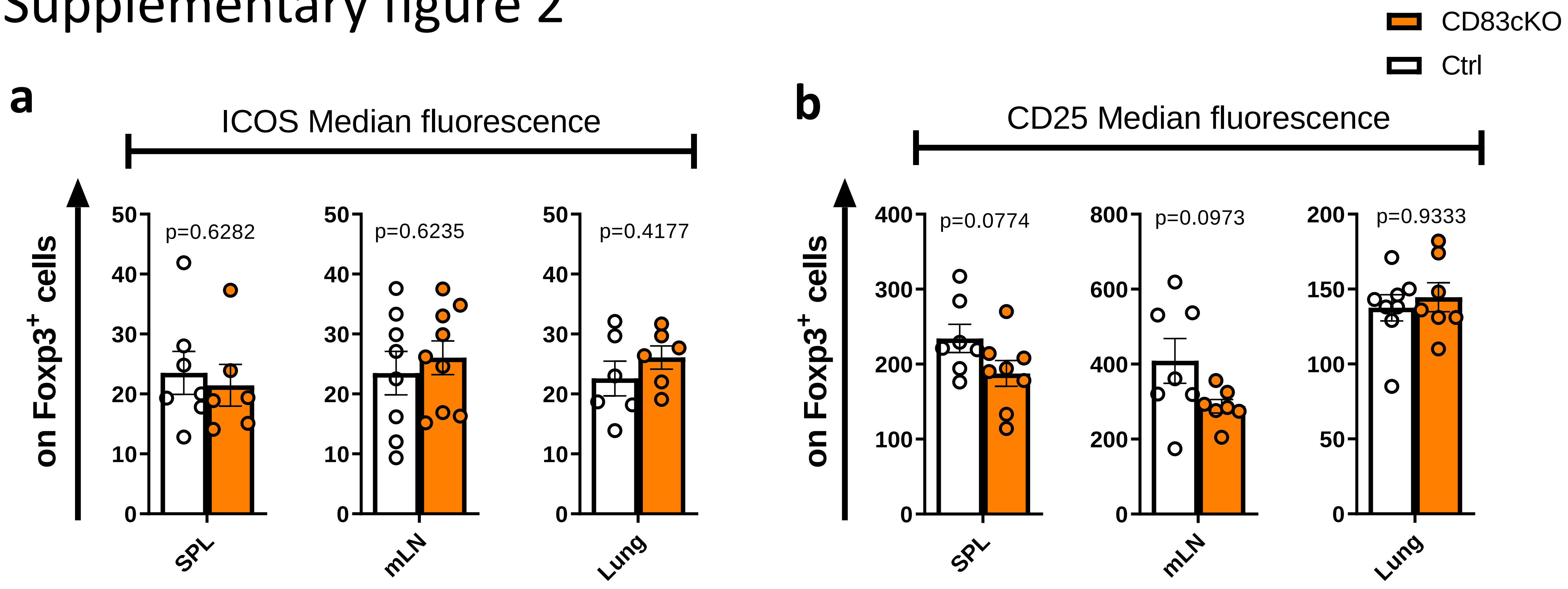

Supplementary figure 2. CD83cKO mice show a higher activatory status and an impaired non-lymphoid-tissue (NLT) function

FACS analysis of splenic (SPL); cervical lymph node-, (cLN) and lung-resident Treg cells ($CD4^+Foxp3^+$) from 8-12 week old mice: surface receptor expression of (a) ICOS and (b) CD25 on Tregs. (Ctrl n=8-12, CD83cKO n=8-12).

Supplementary figure 3

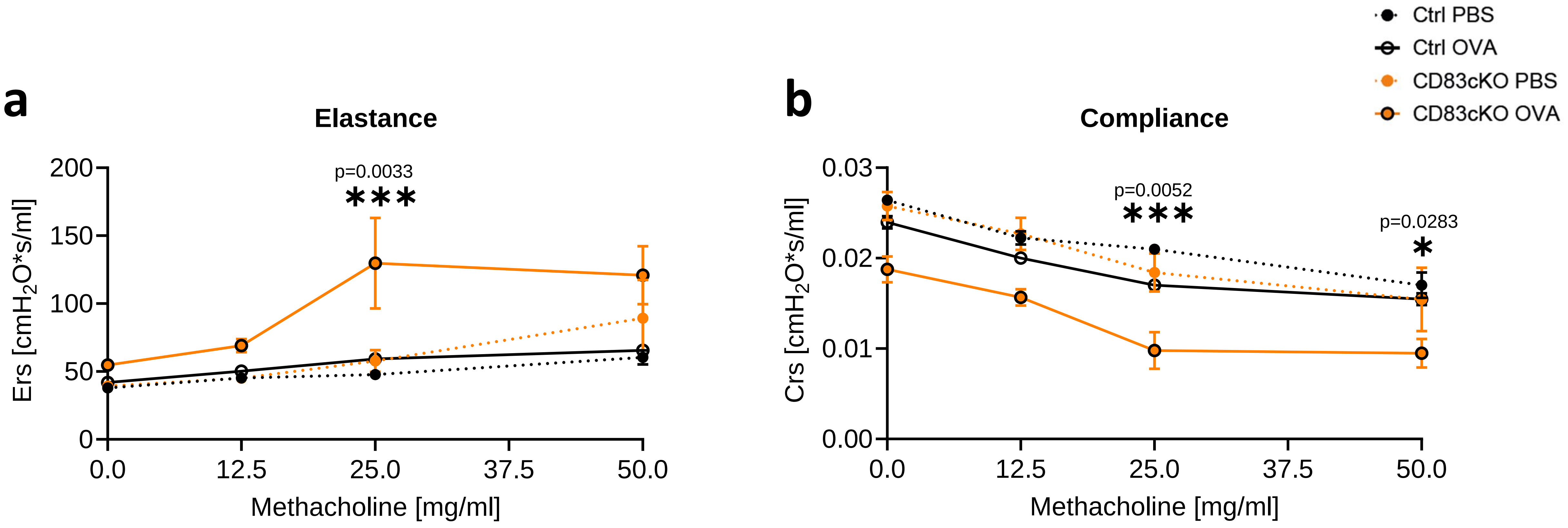

Supplementary figure 3. CD83cKO mice developed an exacerbated AHR and eosinophilia in allergic asthma

(A-B) Invasive AHR analysis is performed to determine the elastance (Ers) and the compliance (Crs) of the lung upon methacholine challenge. 4 experiment groups: PBS control groups and OVA-treatment groups for both genotypes (Cre+/- control wildtypes and CD83cKO) from 8-12 week old mice. (Cre-Ctrl n=4-5, CD83cKO n= 4-5). Statistical analysis was performed using a two-way Anova test with Tukey´s correction test. *p < 0.05, **p < 0.01, ***p < 0.001, ****p < 0.0001. Graphs without stars are considered not significant: (C) 0mg p=0.8632; 12.5mg p=0.6226; 50mg p=0.3346; (D) 0mg p=0.0703; 12.5mg p=0.1705

Supplementary figure 4

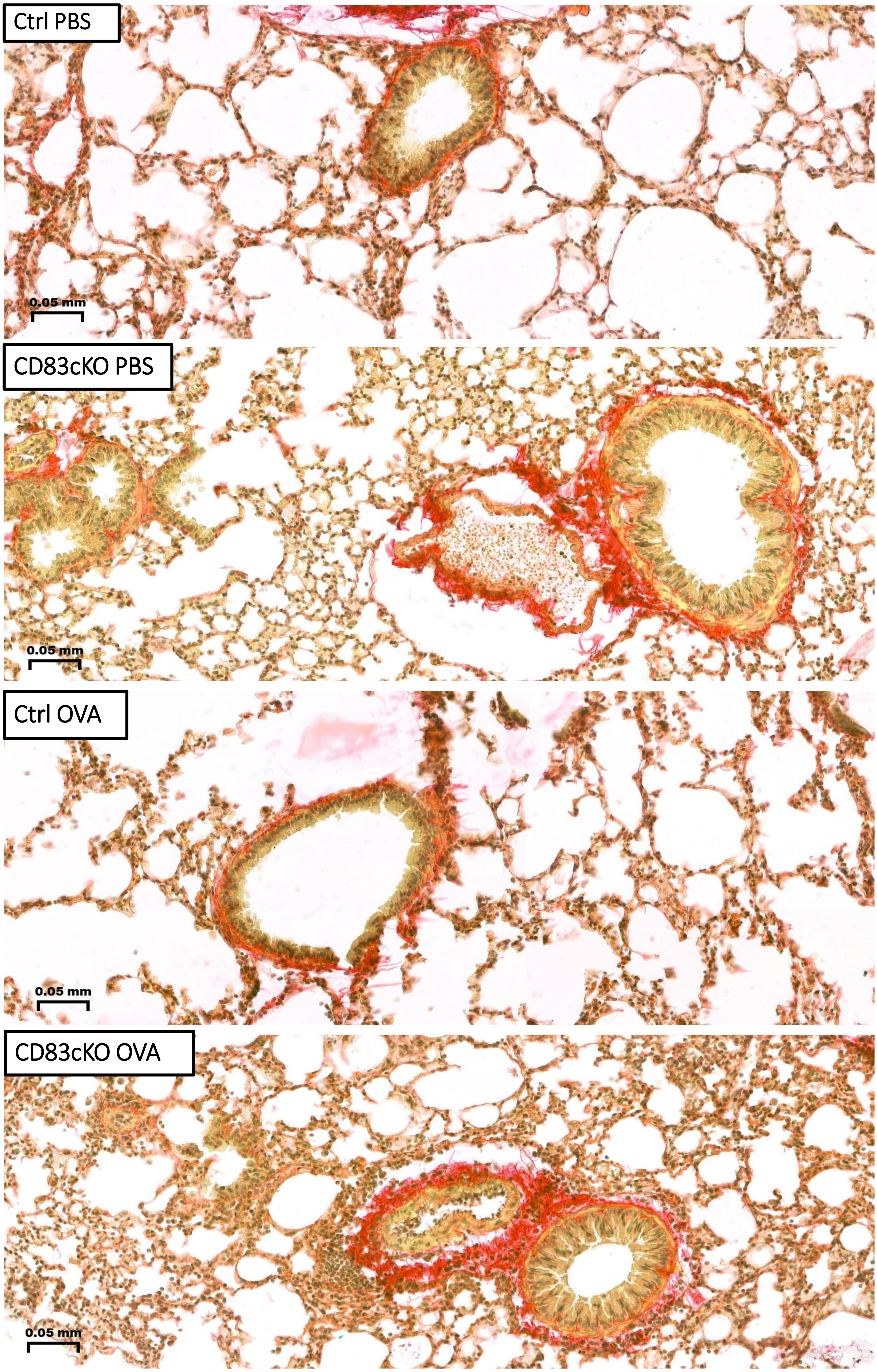

Supplementary Figure 4. CD83cKO mice showed pathological changes in lung morphology

Uncropped histological images shown in Figure 2d.

Supplementary figure 5

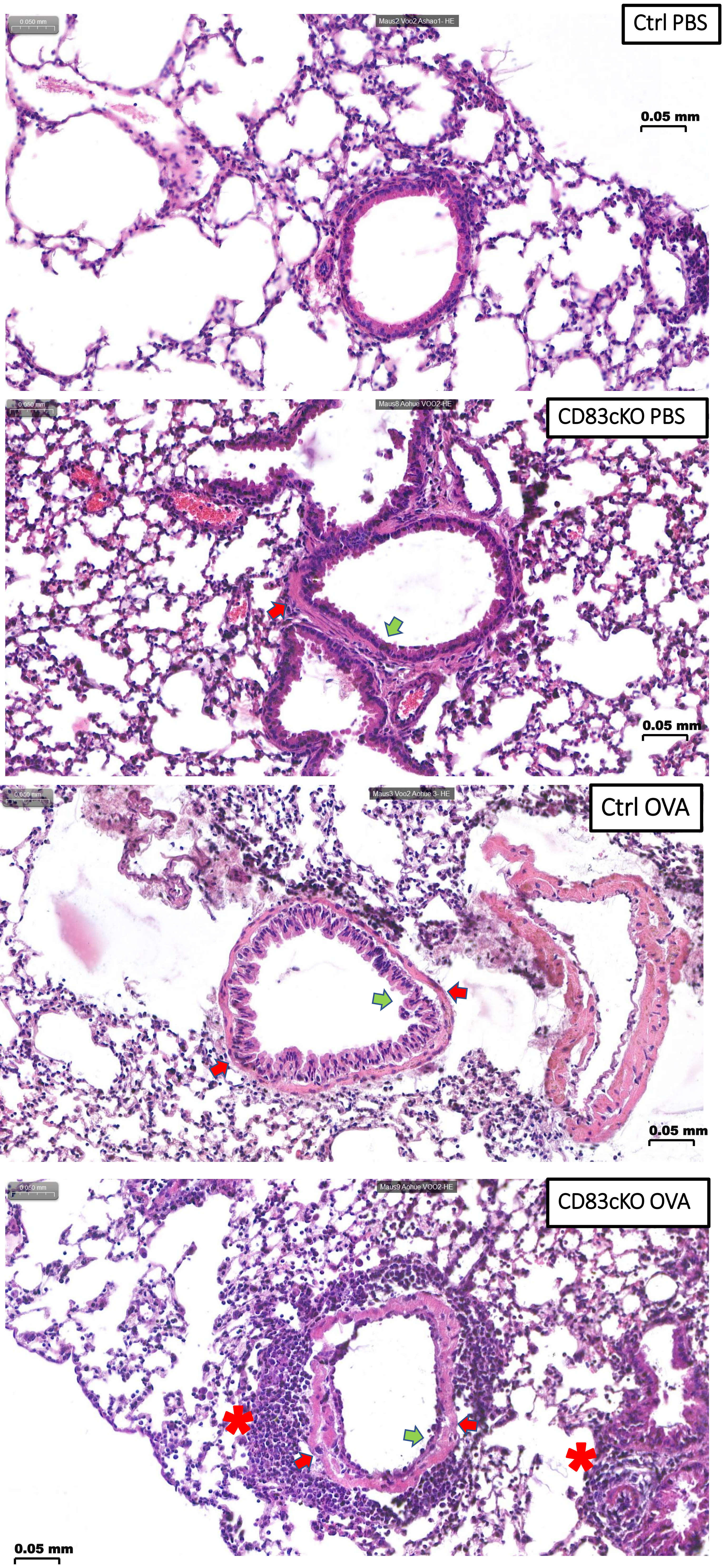

Supplementary Figure 5. CD83cKO mice showed pathological changes in lung morphology

Histological features of naïve and OVA-treated Ctrl WT and CD83cKO mice. Paraffin-embedded, 5 µm thick serial sections of the lung were taken for hematoxylin and eosin (H&E) staining. Red arrows demonstrate smooth muscle tissue changes, green arrows the bronchial epithelium and red stars show immune cell infiltration.

Supplementary figure 6

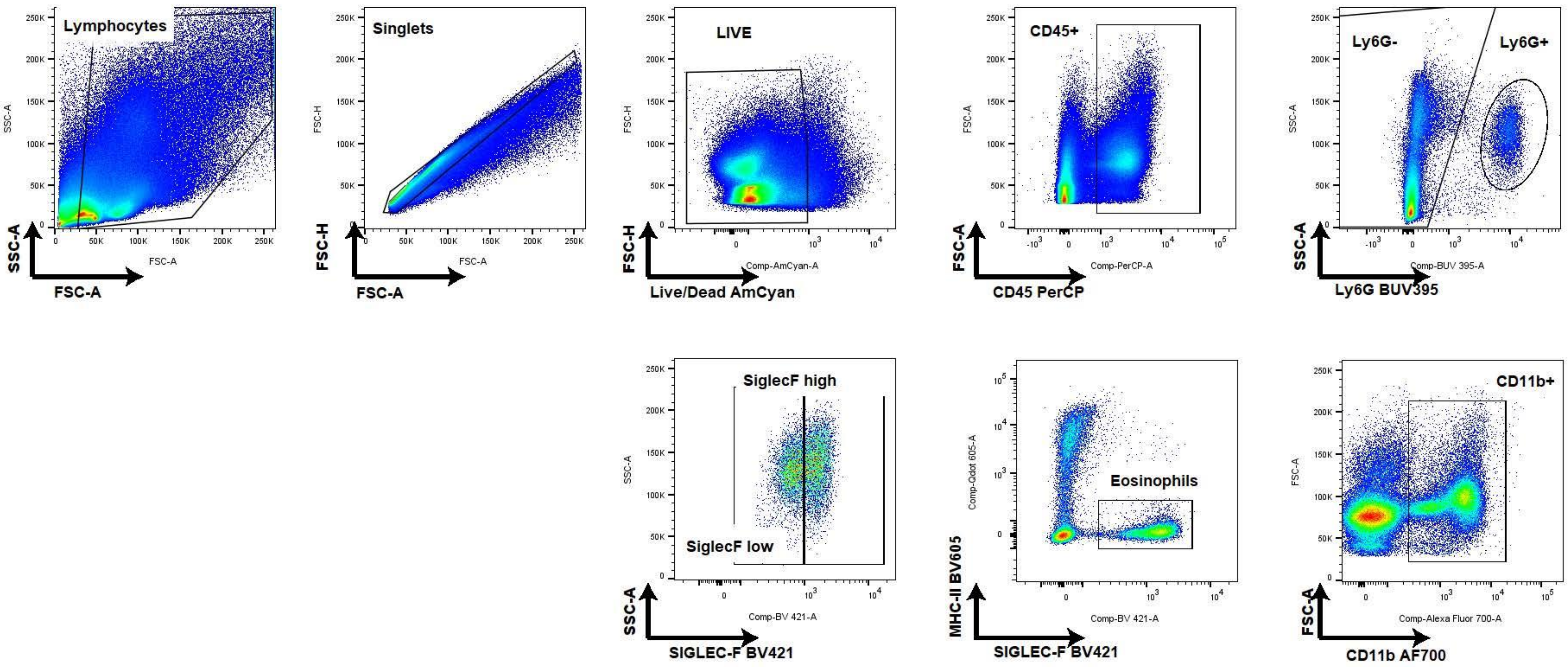

Supplementary figure 6. Flow cytometric gating strategy for the analysis of immigrated eosinophils Gating strategy to identify eosinophils (CD45+Ly6G-CD11b+SiglecF+). Color dot plot of a representative subject. Approximately 100.000 events on CD45+ cells were measured for each sample.

Supplementary figure 7

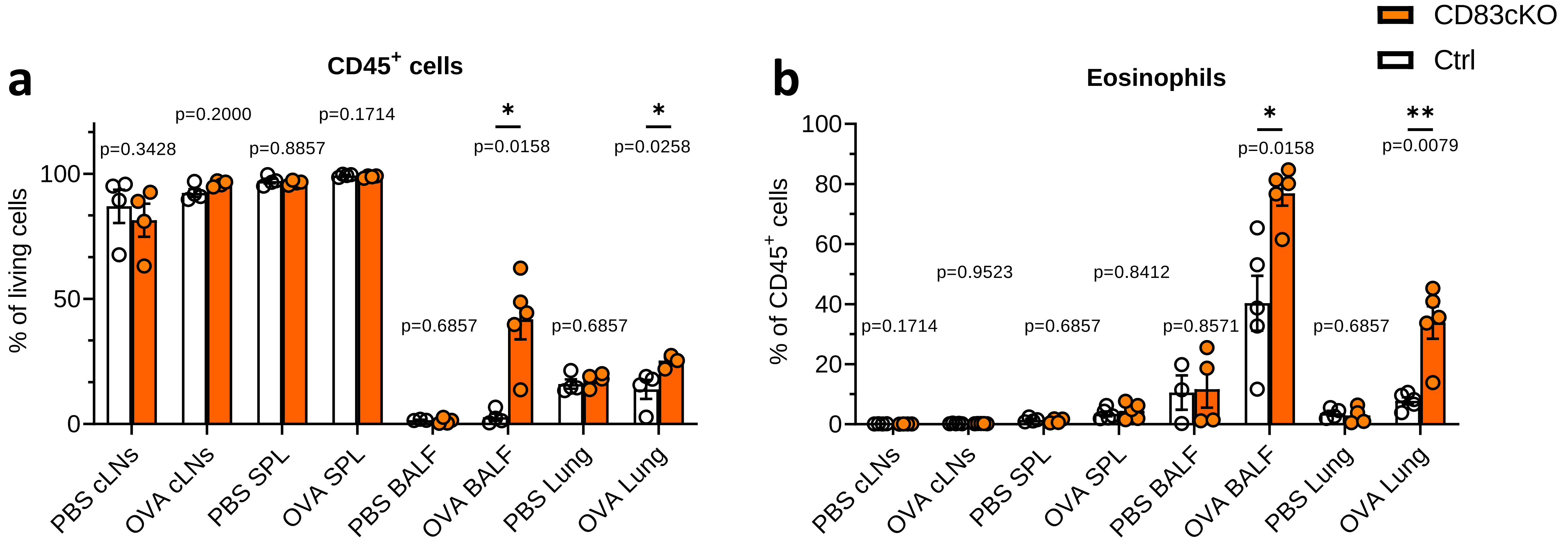

Supplementary figure 7: CD83cKO mice developed an exacerbated eosinophilia in allergic asthma

FACS analysis of splenic (SPL); cervical lymph node-, (cLN) and lung- resident immune cells as well as migratory cells into the bronchoalveolar lavage-fluid (BALF) from 8-12 week old mice. (**a**) frequencies of CD45+ immune cells were analyzed in the different tissues. (**b**) frequencies of immigrated eosinophils are shown amongst all CD45+ cells.

Supplementary figure 8

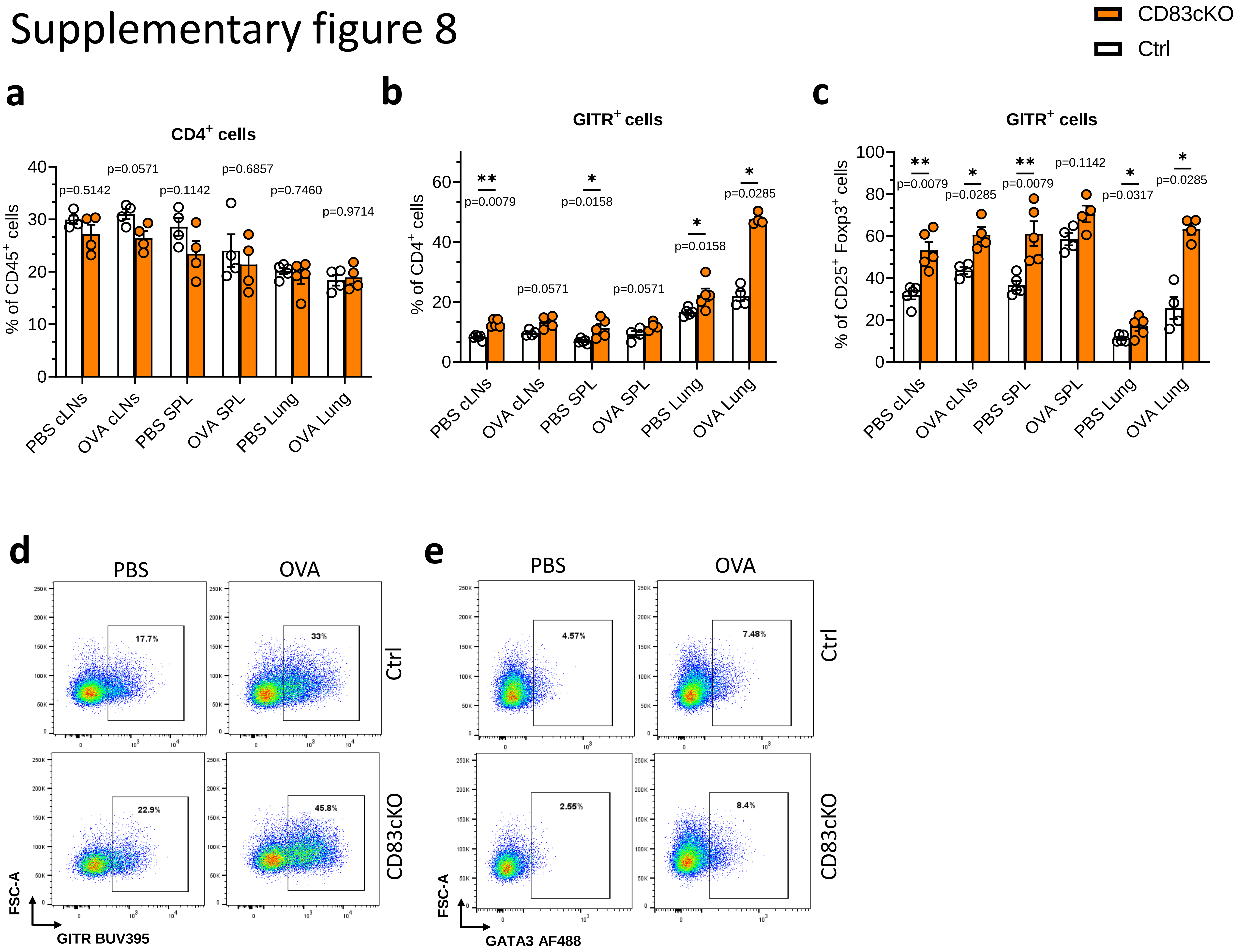

Supplementary figure 8. Asthmatic lungs of CD83cKO mice resulted in enhanced effector T cell responses and a highly activated phenotype

(a-c) FACS analysis of splenic (SPL); cervical lymph node-, (cLN) and lung- resident CD4+ T cells and Tregs from 8-12 week old mice: the frequencies of CD4+ cells are determined **(a);** surface receptor expression analysis of GITR in CD4+ T cells **(b)** and in Tregs **(c)**. 4 experiment groups: PBS control groups and OVA-treatment groups for both genotypes (Cre+/- control wildtypes and CD83cKO) from 8-12 week old mice. (Cre-Ctrl n=4-5, CD83cKO n= 4-5). **(d,e)** Representative plots of CD4+ T cells gated for GITR (**d**) and GATA3 (**e**).

Supplementary figure 9

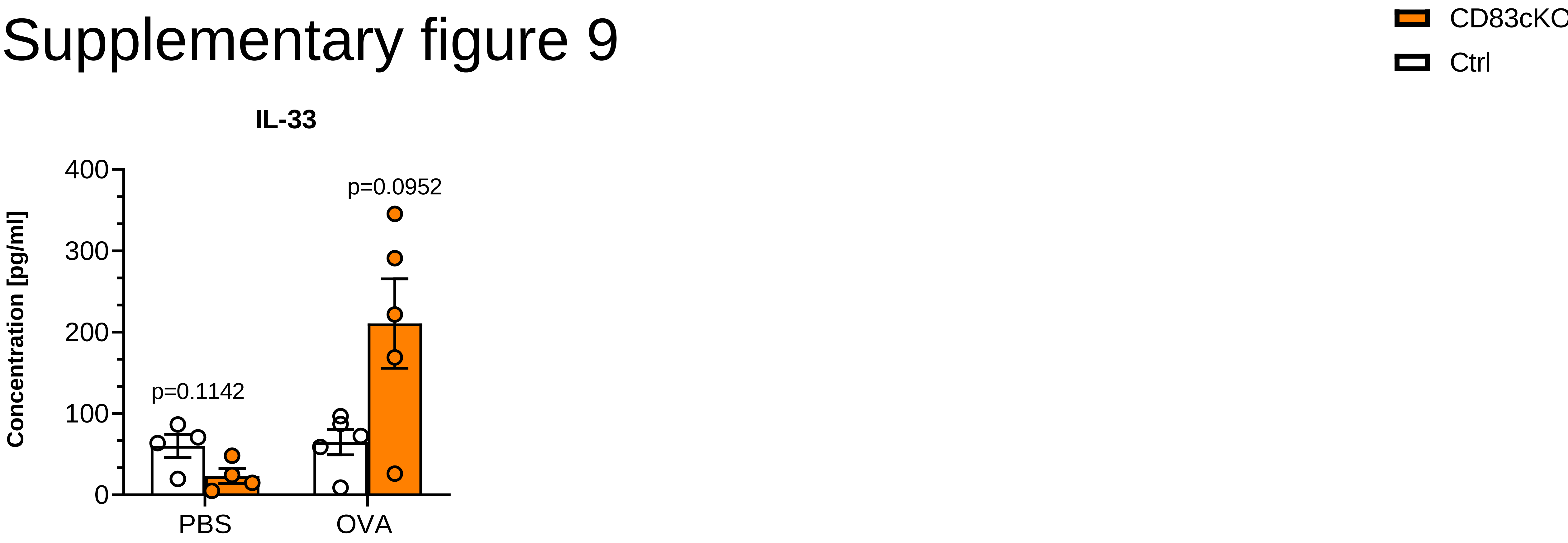

Supplementary figure 9. Circulating levels of IL-33 in asthmatic CD83cKO mice resulted in enhanced levels indicating higher inflammation and tissue injury upon allergic asthma disease

Determination of alarmin IL-33 levels in the sera from 8-12 week old mice using a commercial ELISA test. 4 experiment groups: PBS control groups and OVA-treatment groups for both genotypes (Cre+/- control wildtypes and CD83cKO) from 8-12 week old mice. (Cre-Ctrl n=4-5, CD83cKO n= 4-5).

Supplementary figure 10

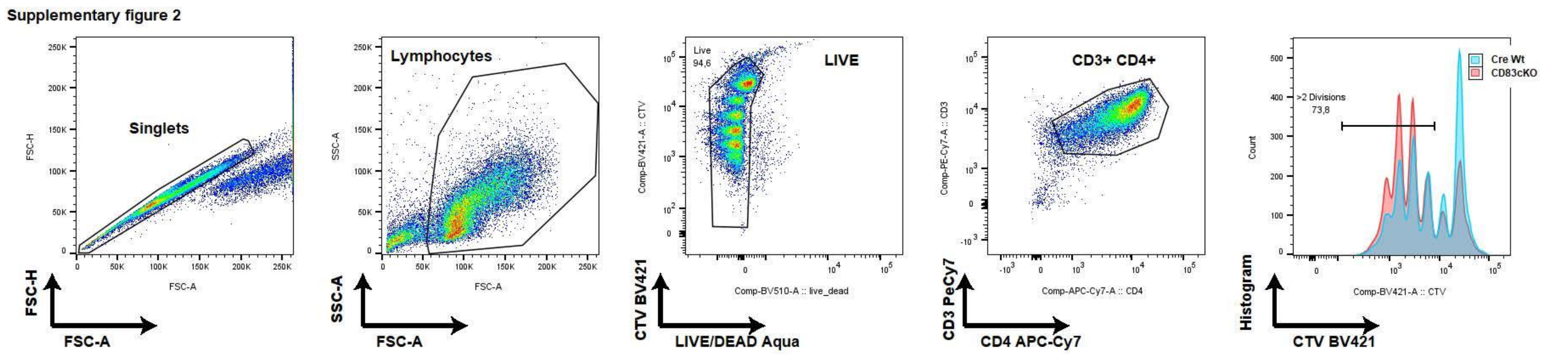

Supplementary figure 10. Flow cytometric gating strategy for the analysis of the proliferation and differentiation of CD4+ T-cell cells Gating strategy to identify CD3+CD4+T-cells. Different surface receptors (CD25 and GITR) and transcription factors (Foxp3 and GATA3) were analyzed on this subset. Color dot plot of a representative subject. CellTrace Violet Cell (CTV) peaks show the numbers of divisons of the cells stimulated with anti-CD3/anti-IFNg/IL4. Approximately 20.000 events on CD3+CD4+ T cells were measured for each sample.

Supplementary figure 11

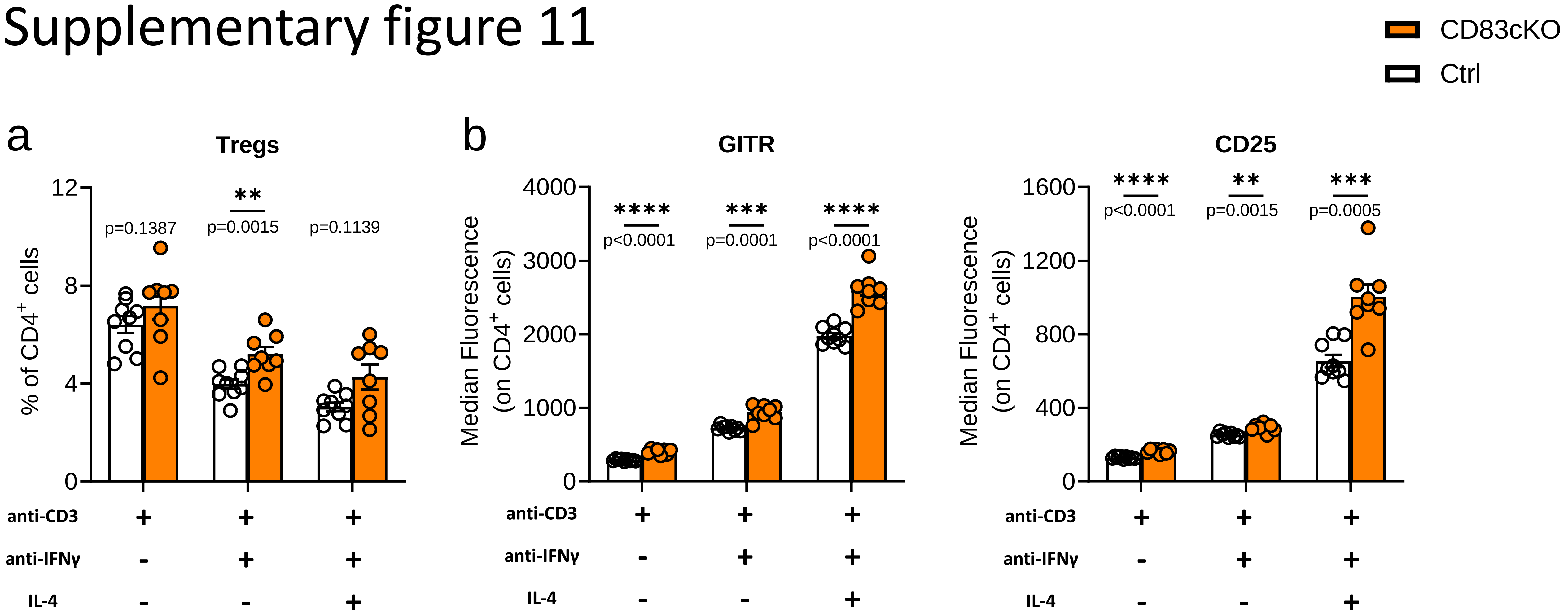

Supplementary figure 11. Flow cytometric analysis of the proliferation and differentiation of CD4+ T-cell cells

LPS-stimulated DCs are co-cultured with CellTraceTMViolet (CTV)-labeled CD4+ T cells from wild-type or CD83cKO mice in the presence of stimuli (anti-CD3/anti-IFNγ/IL-4) for 4 days. (**a**) Percentages of $CD25^{+}FoxP3^{+}$ T cells (Tregs) among all $CD4^{+}$ T cells. (b) Surface display of GITR and CD25 as measured by flow cytometry (n=9, pool of 3 independent experiments).

Supplementary figure 12

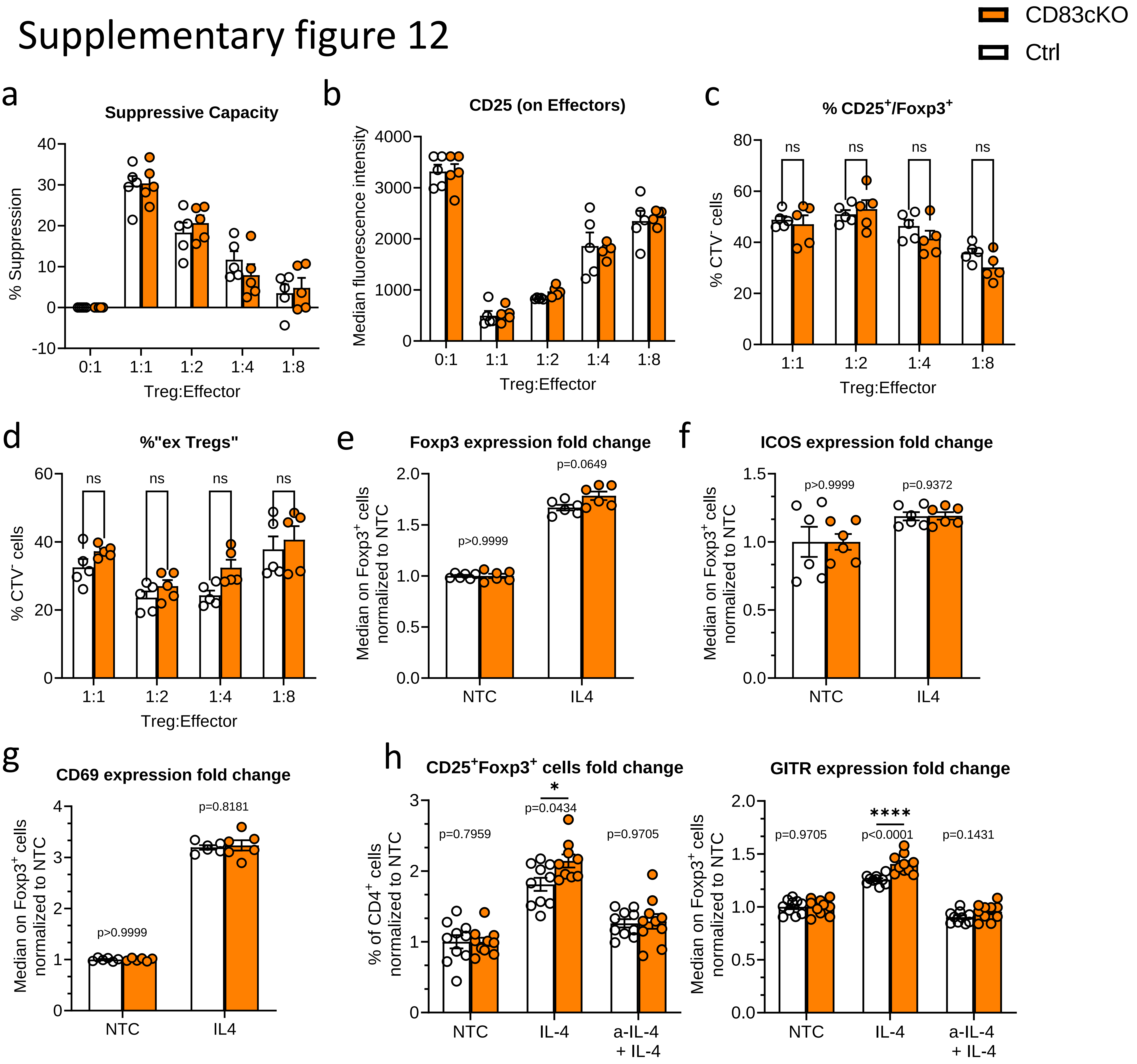

Supplementary figure 12. Tregs of CD83cKO mice have normal suppressive functions but show an enhanced IL-4 responsivness

(a) Suppressive capacity of Tregs when co-cultured with effector T cells under Th2-conditions. **(b)** CD25 on proliferating effector T cells as measured by flow cytometry. Percentage of **(c)** $CD25^+/FoxP3^+$ cells and **(d)** exTregs among CTV^- T cells (n=5). **(e)** flow cytometric analysis of FoxP3 on sorted Tregs stimulated with IL-4, normalized to non-treated control (NTC). Relative increase in surface **(f)** ICOS and **(g)** CD69 in response to IL-4 (n=6). **(h)** FACS analysis of overnight *in vitro* stimulated splenic $CD4^+$ T cells from 8-12 week old mice: Non-treated controls (NTC); IL4; anti-IL4 pre-treated controls (a-IL4+IL4): Frequencies of $CD25^+$Tregs ($CD25^+CD4^+$ $Foxp3^+$) as well as the median fluorescence of GITR were determined on $Foxp3^+$ Tregs (Ctrl n=10, CD83cKO n= 10).
